# The Manipulability Effect in Object Naming

**DOI:** 10.5334/joc.30

**Published:** 2018-05-30

**Authors:** Anna Lorenzoni, Francesca Peressotti, Eduardo Navarrete

**Affiliations:** 1 Università di Padova, IT

**Keywords:** word production, object manipulability, object naming, lexical access, language production

## Abstract

Seeing objects triggers activation of motor areas. The implications of this motor activation in tasks that do not require object-use is still a matter of debate in cognitive sciences. Here we test whether motor activation percolates into the linguistic system by exploring the effect of object manipulability in a speech production task. Italian native speakers name the set of photographs provided by Guérard, Lagacè and Brodeur (*Beh Res Meth*, 2015). Photographs varied on four motor dimensions concerning on how easily the represented objects can be grasped, moved, or pantomimed, and the number of actions that can be performed with them. The results show classical psycholinguistic phenomena such as the effect of age of acquisition and name agreement in naming latencies. Critically, linear mixed-effects models show an effect of three motor predictors over and above the psycholinguistic effects (replicating, in part, previous findings, Guérard et al., 2015). Further research is needed to address how, and at which level, the manipulability effect emerges in the course of word production.

## Introduction

When presented with a picture of a ‘chair’ that we have never seen before, we have no difficulty in correctly naming the picture as “chair” in less than one second. In recent decades much effort has been put into describing how information is processed from a perceptual stimulus (a particular image) through to linguistic content (the word associated to the image). As a result of these efforts, researchers have identified some of the variables that modulate the speed and the accuracy with which we retrieve words from our mental lexicon during object naming tasks. These are, among others, the visual properties of the image, the semantic aspects associated to the corresponding concept, as well as the lexical and phonological characteristics of the target word. Here we focus on a variable that has received less attention in the field of word production, that is, the manipulability of the object represented in the image. Broadly speaking, object manipulability refers to any motor dimension associated to an object that recruits the way we can interact with that object, such as for instance how an object can be grasped, or the action we perform when using that object (see below for more details).

It is well documented that the mere vision of a tool automatically triggers activation of brain regions that encode information about motor-based properties of the object (Chao & Martin, 2000). In addition, the dorsal visual pathway is differently involved depending on object manipulability and the information provided by the dorsal stream can be used during the categorization of manipulable objects (see e.g., Almeida et al. 2008). Here we explore whether differences on object manipulability can affect language production as well. Initial evidence in this direction comes from the study conducted by Witt and colleagues (2010). Participants in that study were required to name pictures belonging to two semantic categories, tools and animals, while simultaneously squeezing a ball with one hand. The tool and animal images could be oriented to the right or to the left, while participants could be squeezing the ball with their right or left hand. Witt and colleagues reported faster naming latencies and more accurate responses in naming the tools that were orientated with the handle facing away from the squeezing hand than with the handle facing towards the squeezing hand, while no differences were observed in naming images of the animal category. This pattern was interpreted as evidence that motor simulation has a functional role in tool identification. This conclusion however is questioned by other recent studies reporting a manipulability effect even when no hand movement is performed and with a set of objects that do not contain a handle element but can be easily grasped. In a standard naming task Salmon, Matheson and McMullen (2014) reported faster naming latencies for manipulable objects (e.g., ruler) than for non-manipulable objects (e.g., stove) (see for discussion, Matheson et al., 2014).

Further evidence comes from the study by Guérard, Lagacè and Brodeur (2015). In that study, 560 objects were rated on four dimensions of manipulability by four different groups of participants. A 7-point Likert scale from 1 (difficult) to 7 (easy) was used to rate how difficult/easy each object was to grasp, move, and pantomime for the Grasp, Move, Pantomime scales, respectively. In addition, the number of actions that could be performed with each object was rated using a scale from 0 (no actions) to (6+), more than 6 actions. A new group of participants named all the objects. A hierarchical multiple regression analysis showed that objects that were associated to a high number of actions and that were easy to grasp and pantomime were named faster, while those that were easy to move were named slower.

The reviewed evidence suggests that motor properties associated to the target object (what we called, object manipulability) might affect word production. It is still unclear, however, to what extent manipulability either facilitates or interferes with naming latencies; and the role of hand movements for the emergence of the phenomenon. In this data report we aimed at replicating the object naming experiment conducted by Guerard and colleagues. For this we had three motivations. First, we jointly consider the effect of some critical variables known to affect word production that were not controlled in the study by Guerard and colleagues. Specifically, we took into account the features of the phonological neighborhood and the estimated age of acquisition of each image word. Second, Guerard and colleagues analyzed their data using regression analyses on mean latencies. Here we analyzed naming latencies and error rates using mixed effects regression models performed at the single trial level, providing a more fine-grained approach in comparison to traditional regression techniques. Basically, mixed models allowed us to test the influence of all the selected predictors on the dependent variables, taking into account both by-participants and by-item variability (Baayen et al., 2008). And third, replicability has been recently highlighted as a critical research issue in cognitive science. We aim therefore at assessing the reliability of the manipulability effect by testing it in a language never tested before, that is, Italian.

## Method

*Participants*. Twenty five native Italian speakers took part in the experiment (22 females, 3 left-handed; mean age = 20.6, SD = 1.84).

*Materials*. The set of 560 normed stimuli published by Guérard and colleagues (2015) was selected as experimental material for the naming task. For each photograph we considered the level of familiarity and visual complexity ratings provided by Brodeur and colleagues (2010) and the four different manipulability ratings provided by Guerard and colleagues.

*Procedure*. An experimental trial consisted of the following events. A fixation cross was shown in the center of the screen for 500 ms followed by a blank screen for 500 ms. Following the blank screen the target picture was presented for 3000 ms or until the participant’s response. Participants were asked to name the object as quickly and accurately as possible. They were instructed to avoid the use of determinants (e.g., the, a) or any kind of adjective (e.g., color, size). If they did not know the name of the object, they were instructed to remain silent. Response latencies were measured from the onset of the picture. The next trial began 1500 ms after the onset of participants’ response. Stimulus presentation and response recording were controlled by DMDX program (Forster & Forster, 2003). The set of items was presented in a different random order for each participant. There was a short pause of 60 seconds after 70 trials. The first six trials were warm-up trials containing 6 filler images.

## Analysis

The influence of object manipulability was explored within a subset of images with a name agreement value equal to or above 50%. This criterion was selected in order to exclude the possible impact of poor visual structural descriptions of the target stimuli, and the impact of idiosyncratic linguistic characteristics of the target words (see Barry et al., 1997). Furthermore, the original set of Guerard and colleagues might contain more than one exemplar of the same concept (e.g., there were 4 different exemplars of the item *bottle*). In these cases, we selected the item with the highest value of H statistic. In sum, analyses were performed using a set of 7100 trials corresponding to 284 different items (206 man-made objects and 78 natural objects). Here we present the results of the analyses on the man-made items (e.g., vehicles, tools) since they constitute the category where a manipulability effect is expected (for item properties see Table S1 in Supplemental Materials). Analyses on natural objects (e.g., animals, body-parts) are reported in the Supplemental Materials. Analyses were performed with the R statistical software (R Core Team, 2016).

Before testing the role of object manipulability in the selected set of 284 items, we controlled for the effect of several variables that previous research have identified as critical predictors of object naming performance:

- Visual complexity (from Brodeur et al., 2010). This variable refers to the degree of image complexity in terms of the quantity of details and the intricacy of the lines on a 5-point scale (1-very simple, 5-very complex image).
- Familiarity (from Brodeur et al, 2010). This variable refers to the degree to which people come in contact or think about the concept on a 5-point scale (1-very unfamiliar; 5-very familiar).
- Age-of-Acquisition (AoA). AoA values have been estimated for each image by 24 new Italian native speakers who did not participate in the main experiment (mean age = 20.6, SD = 1). Participants rated the age at which they thought they had first learned each word on a 1–13 Likert scale (1 = learned at 0–1 year, 13+ = learned at age 13 or after, with 1 year age bands in between).
- H statistic (as index of agreement). H is a logarithmic function describing the different names that an object receives and the proportion of participants giving each name (Snodgrass and Vanderwart, 1980). A picture that elicited the same name from every participant in the sample who was able to name it has an H value of 0. Increasing H value indicates decreasing name agreement and, generally, decreasing percentages of participants who all gave the same name. H statistic was calculated on the experimental data.
- Word frequency (from the PhonItalia database, Goslin et al., 2014). A logarithmic transformation was applied to avoid the undue influence of extreme values in the regression.
- Word length measures. NumPhones (number of phonemes) and NumSyll (number of syllables).
- Phonological predictors (from the PhonItalia database, Goslin et al., 2014). Density of the phonological neighborhood (Phon_N), mean log frequency of the phonological neighborhood (Phon_N_MFreq), phonological Levensthein distance (PLD).

*Data Analysis*. Onset latency and accuracy were checked offline using the CheckVocal software (Protopapas, 2007). Production of clearly erroneous picture names and verbal dysfluencies (stuttering, utterance repairs) were considered as errors and removed from naming latencies analysis (10.1%, see Supplemental Materials). Following previous studies that analyze the impact of several predictors on behavioral data sets (e.g., Sadat et al., 2014; Scaltritti et al., 2016), analyses were run in two steps. In the first step we assessed the correlations among predictors through a hierarchical clustering analysis using the *varclus* function of the “Hmisc” package (Harrell, 2017). This allowed us to identify clusters of predictors (i.e., predictors with a Spearman similarity coefficient > .5). In order to select the more important predictor within each cluster, we ran a random forest analysis on naming latencies using the function *cforest* of the package “party” (Hothorn et al., 2006). Predictors that resulted to have the highest measure of variable importance within each cluster were selected as representing that cluster and further analyzed. In the second step, we analyzed latencies of correct responses with linear mixed models (LMM) and accuracies with generalized linear mixed models (GLMM) using the package “lme4” (Bates et al., 2015). In order to ensure that any effect of object manipulability was significant over and above the variation explained by other predictors, we first built a Control Model containing all control predictors that yield a better fitting model of the data. We then tested each of the four manipulability variables separately against the Control model. For model comparison we performed the likelihood ratio test and took into consideration the Bayesian Information Criterion (BIC; Schwarz, 1978). We calculated the differences between the preceding model and the new one including a new predictor (Δbic). A positive Δbic value implies that a given model is better than the previous one. We calculated the Bayes Factor›s (BF) approximation using the formula exp(Δbic/2) (Raftery, 1995); using BF we are able to compare the relative evidence of a model. In general, the higher the Δbic and the BF are, the more evident the model is with respect to the other model.

## Results

**Figure 1:**
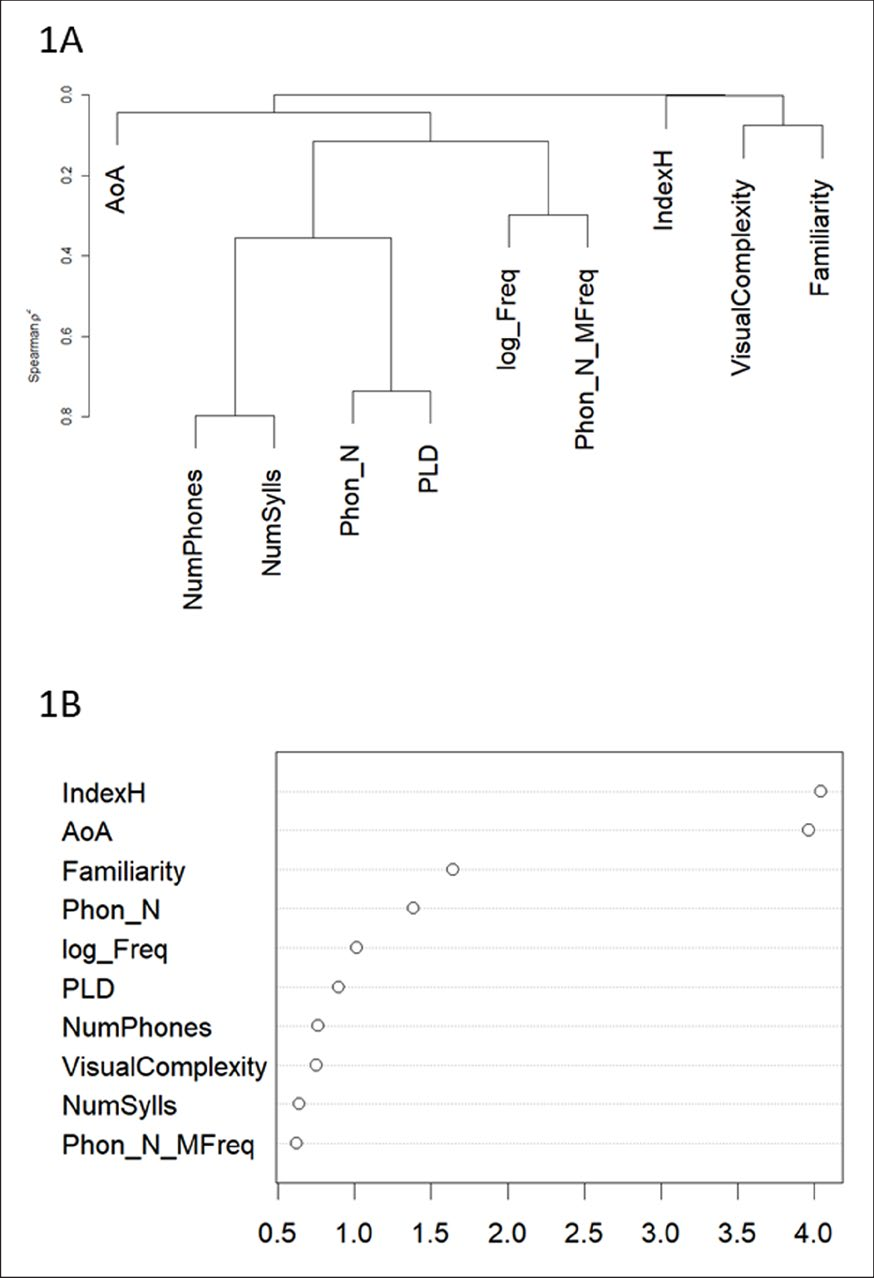
**A:** Hierarchical clustering analysis using Spearman’s p² for naming latencies within man-made items. **B:** Variable importance plot obtained through random forest analysis for the same set of predictors within man-made items.

*Predictor selection*. Figure 1A shows a graphical representation of the correlation structure among all the control predictors. Two clusters of highly correlated predictors emerged. In order to select the most important predictor from each of the two sets, we ran the random forest analyses four times to ensure the results were stable. The same outcomes were obtained in all four analyses (Figure 1B shows the results of the first random forest analysis). Based on this outcome, we retained NumPhones and Phon_N as predictors since they were consistently ranked higher than NumSyll and PLD, respectively.

*Naming latencies*. Parameters were estimated using LMM. As the data were not normally distributed, we used the Box-Cox test (Box & Cox, 1964), using the function boxcox in the package “MASS” (Venables & Ripley, 2002) to estimate the most appropriate transformation for the data to reduce skewedness and approximate a normal distribution. The test indicated that the reciprocal transformation was the most appropriate transformation (we used instead –1000/RT to facilitate reading of the results).

The null model (i.e., M0) contained random intercepts for participants and items only and no predictors. We then added single predictors as fixed effects incrementally, with a new model for each predictor. The order with which control predictors are included in the model paralleled the cognitive processes involved in object naming, that is, from perceptual stimulus processing to the linguistic response. Thus, we explored in this order, Visual complexity, Familiarity, H statistic, AoA, log_Frequency, NumPhones, Phon_N and Phon_N_MFreq. We kept for further analysis only those predictors that produced a significant increase in the explained variance in comparison to the preceding model. Any predictor that did not produce an increase in the explained variance was dropped from further analyses. Direct comparison between M0 and M1 showed that the inclusion of the predictor Visual complexity did not improve the model fit. The comparison between M0 and M2 showed that the inclusion of the predictor Familiarity explains the data 10 times better than M0. Items with high familiarity ratings elicited faster naming latencies than items with low familiarity ratings. We kept Familiarity as a critical predictor and explored the influence of the other variables. Before examining the effects of lexical predictors, we included in the model the H statistic in order to account for the variability due to alternative correct responses given to the same stimulus. The inclusion of the H statistic (M3) produced higher Δbic values in comparison to M2 and was kept as a critical predictor. Items with higher H produced faster naming latencies. The inclusion of the predictor AoA (M4) also increased the explained variance and was kept as a critical variable. Early acquired items were named faster than late acquired items. Log Frequency, failed to increase the model fit and was not included in the model. Finally, the inclusion of none of the phonological variables (NumPhon, Phon_N and Phon_N_MFreq) improved the model fit. These predictors were therefore not included in the Control model (see Table 1). Note that although the inclusion of the predictor Phon_N in M7 was significant, χ^2^ = 6.33, p = .01, its inclusion does not yield an overall better fitting model compared to M4. In sum, the Control model we used to investigate the manipulability effect included the effects of Familiary, H statistic and AoA.

**Table 1:** The fit indices on the naming latencies analysis on control predictors for man-made items.

Model	Fixed effects	Model Df	*χ*^2^	P	BIC	Δbic	Approx. BF

M0		4			1119		
M1	Visual complexity	5	<.01	=.99	1127	–8.43	.014
M2	Fam	5	13.09	<.001	1114	4.65	10.27
M3	Fam + H	6	54.57	<.001	1068	46.13	>10000
**M4 (Control)**	**Fam + H + AoA**	**7**	**42.63**	**<.001**	**1034**	**34.19**	**>10000**
M5	Fam + H + AoA + Log_Freq	8	0.06	=.79	1042	–8.37	0.015
M6	Fam + H + AoA + NumPhones	8	1.61	=.2	1041	–6.82	0.033
M7	Fam + H + AoA + Phon_N	8	6.33	=.011	1036	–2.11	0.34
M8	Fam + H + AoA + Phon_N_MFreq	8	1.52	=.21	1041	–6.82	0.031

*Note:* Df = degree of freedom; P = probability value; BIC = Bayesian Information Criterion; Δbic = differences between the last model that displayed a significant increase in terms of explained variance and the current model; Approx. BF = Bayes Factor’s (BF) approximation, exp(Δbic/2). Fam = Familiariy. Log_Freq = log word frequency. In Bold the Control Model.

**Figure 2:**
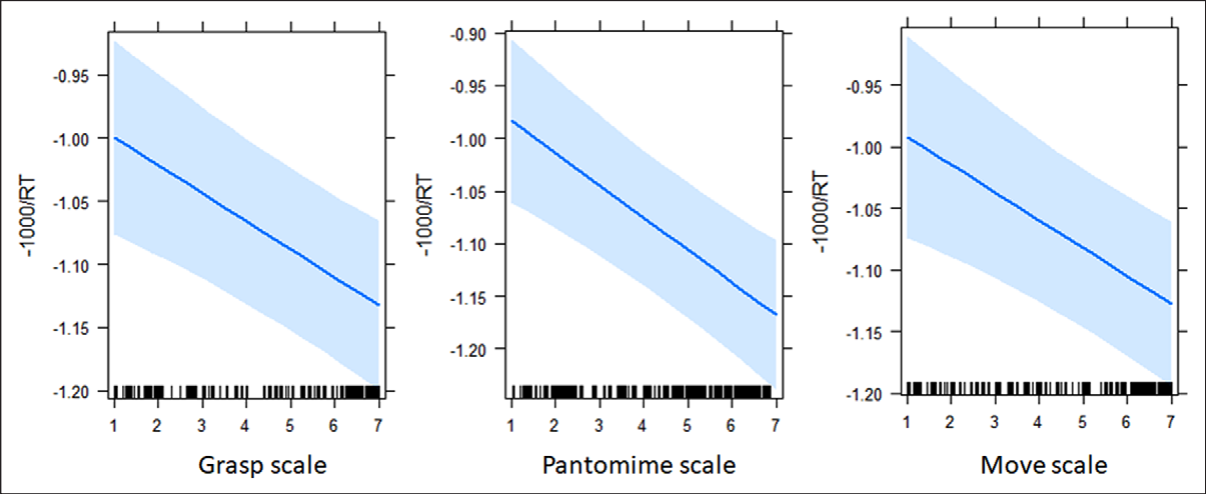
Effects of the predictors Grasp, Pantomime and Move on the naming latencies.

We then tested separately the four object manipulability variables against the Control model. As can be seen in Table 2, the predictors Grasp, Pantomime and Move increased significantly the variance explained. Items with higher Grasp, Pantomime and Move rates were named faster (see Figure 2). The inclusion of the predictor Number of Actions did not yield an overall better fitting than the Control model (see Table 3).

**Table 2:** The fit indices on the naming latencies analysis on manipulability predictors for man-made items. NumActions = Number of Actions.

Model	Fixed effects	Model Df	Chisq	P	BIC	Δbic	Approx. BF

**Control**	**Fam + H + AoA**	**7**					
Grasp	Fam + H + AoA + Grasp	8	17.587	<.001	1025	9.13	96.39
Pantomime	Fam + H + AoA + Pantomime	8	22.56	<.001	1020	14.12	1168.72
Move	Fam + H + AoA + Move	8	14.44	<.001	1028	6.01	20.15
NumActions	Fam + H + AoA + NumActions	8	6.38	=.011	1036	–2.057	0.35

**Table 3:** The fit indices on the Accuracy analysis for man-made items.

Model	Fixed effects	Model Df	Chisq	P	BIC	Δbic	Approx. BF

M0		3			3103		
**Control**	**Fam + H + AoA**	**6**	**72.57**	**<.001**	**3056**	**46.96**	**>1000**
Grasp	Fam + H + AoA + Grasp	7	6.02	=.014	3058	–2.52	0.283
Pantomime	Fam + H + AoA + Pantomime	7	5.46	=.019	3059	–3.08	0.214
Move	Fam + H + AoA + Move	7	3.99	=.045	3060	–4.54	0.102
NumActions	Fam + H + AoA + NumActions	7	3.52	=.061	3061	–5.02	0.081

*Accuracy*. For the accuracy analyses the same procedure was followed. The Control model paralleled the one obtained in the latency analyses and contained the predictors Familiarity, H statistic and AoA. By contrast, in the accuracy analyses the inclusion of none of the manipulability predictors yielded a better fit in relation to the Control model.

## Discussion

We conducted an object naming task on 560 items. 206 man-made items with name agreement higher than 50% were analyzed using linear mixed-effects models. Standard psycholinguistic phenomena, such as familiarity and age of acquisition, were observed. Furthermore, the results showed significant effects of three variables related to the manner in which we can interact with the objects (grasping, pantomime, and move). The facilitation effect on naming latencies of grasping and pantomime predictors replicated the findings reported by Guérard and collagues (2015). By contrast, the facilitation effect of the Move scale diverged from Guerard and colleagues’ study. Critically, these effects emerged once all other psycholinguistic variables were taken into consideration in the constructed linear mixed models. Finally, no manipulability effects were reported in the natural category of items (see Supplemental Materials).

To conclude, our data suggest that object manipulability is a critical variable affecting object naming. How does object manipulability influence word production? One possibility is to localize the phenomenon during visual processing. Indeed, a large body of literature shows differences between manipulable and non-manipulable objects during visual identification (Almeida et al., 2008; see also Harris et al., 2012). Another possibility is to describe the phenomenon as a kind of motor priming. If the visual presentation of a manipulable object activates brain areas associated with the motor properties of that object (Chao & Martin, 2000), it might be hypothesized that this activation would spread to the motor regions involved in speech articulation. Further research needs to address these possible interpretations of the manipulability effect in object naming.

## Data Availability

Data file and analyses have been made publicly via the Open Science Framework and can be accessed at https://osf.io/kuw6s/.
